# Supplementary material for: Postural test to differentiate primary aldosteronism from low-renin hypertension: a retrospective single-center study
Source: J Endocrinol Invest. 2025 Nov 18;49(3):609–20. doi: 10.1007/s40618-025-02752-9 (PMC13018078; doi:10.1007/s40618-025-02752-9)
Supplement: Supplementary file 1 — Supplementary Material 1 [file 40618_2025_2752_MOESM1_ESM.pdf]

Postural test to differentiate primary aldosteronism from low-renin hypertension: a retrospective single center study

Authors: Irene Tizianel[1,2], Elena Pagin[1,2], Eugenio Ragazzi[3], Alberto Madinelli[1,2], Simona Censi[1,2], Chiara Sabbadin[1,2], Franco Mantero[2], Caterina Mian[1,2], Mattia Barbot[1,2], Giorgia Antonelli[2,4], Filippo Ceccato[1,2]

Affiliations:

[1] Endocrine Disease Unit, University-Hospital of Padova, Padova, Italy

[2] Department of Medicine DIMED, University of Padova, Padova, Italy

[3] Studium Patavinum, University of Padova, Padova, Italy

[4] Laboratory Medicine Unit, University Hospital of Padova, Padova, Italy

Supplementary material

Supplementary Table 1: Endocrine and clinical characteristics in patients with Primary Aldosteronism (PA) or low-renin hypertension (HTN) according to the renin response during postural stimulation test. CP: clinostatic position, OP: orthostatic position.

	**PA**			**Low renin-HTN**		
	**Always suppressed** (n 54)	**From suppressed to measurable** (n 11)	**Always measurable** (n 15)	**Always suppressed** (n 2)	**From suppressed to measurable** (n 45)	**Always measurable** (n 63)
**Aldosterone baseline** (pmol/L)	764 (539.8-1116.8)	722 (497.8-995.8)	1316 (906.3-1757)	426 (378-558)	441 (366-522)	534 (457-746)
**Renin baseline** (suppressed/tot)	39/54 (72%)	3/11 (2.7%)	4/15 (26%)	2/2	20/45 (44%)	7/63 (11%)
**K^+** (mmol/L)	3.1 (2.8-3.6)	3.2 (2.8-3.3)	3.1 (2.9-3.4)	3.6 (3.5-3.9)	3.9 (3.7-4.1)	3.9 (3.6-4.3)
**Renin in CP** (suppressed/to)	54/54	10/11 (90%)	0/15	2/2	45/45	0/63
**Renin in OP** (suppressed/tot)	54/54	0/11	0/15	2/2	0/45	0/63
**Renin response** (always suppressed/tot)	54/54	1/11 (0.09%)	0/15	2/2	0/45	0/63
**Aldosterone in CP** (pmol/L)	532 (379-838.5)	562 (361.8-1090)	668 (511-1036)	235 (165-325)	216 (168-281)	302 (185-398)
**Aldosterone in OP** (pmol/L)	824 (542-1177)	1060 (896-1270)	1252 (806.5-1769)	512 (470-554)	449 (364-675)	663 (496-798)
**PA subtype**	10 unilateral-3 bilateral	3 unilateral - 1 bilateral	1 unilateral	-	-	-

Supplementary Table 2: Diagnostic test accuracy of aldosterone and renin during postural stimulation test: optimal cut-off values, sensitivity, specificity, and overall accuracy based on ROC analysis. CP: clinostatic position, OP: orthostatic position.

	**AUC (IC 95%)**	**Optimal threshold**	**Sensitivity (%)**	**Specificity (%)**	**Accuracy (%)**
**Aldosterone - CP**	0.87 (0.83–0.93)	164 ng/dL	98.8	83.6	90
**Aldosterone - OP**	0.71 (0.64–0.78)	377 ng/dL	97.5	20	60
**Renin - CP**	0.69 (0.62–0.77)	2.5 ng/mL/h	55	82	68
**Renin - OP**	0.84 (0.78–0.90)	3.5 ng/mL/h	85	71	80
